# The Effect of Clenching and Occlusal Instability on Body Weight Distribution, Assessed by a Postural Platform

**DOI:** 10.1155/2019/7342541

**Published:** 2019-06-23

**Authors:** Konstantinos X. Michalakis, Savvas N. Kamalakidis, Argiris L. Pissiotis, Hiroshi Hirayama

**Affiliations:** ^1^Department of Prosthodontics, Aristotle University Faculty of Health Sciences, School of Dentistry, 54124 Thessaloniki, Greece; ^2^Division of Postgraduate Prosthodontics, Tufts University School of Dental Medicine, Boston, MA 02111, USA; ^3^Graduate Prosthodontics, Department of Restorative Sciences and Biomaterials, Boston University Henry M. Goldman School of Dentistry, Boston, MA 02118, USA

## Abstract

The purpose of this research project was to investigate whether or not clenching and occlusal instability of Angle's Class I malocclusion have an effect on body weight distribution in healthy adult subjects. Twenty adults (fourteen males and six females, ages 27-40, mean age 31.7 years, SD 3.32) were included in this study. The MatScan (Tekscan Inc., Boston, MA) system was used to measure the body weight distribution changes of the subjects. Four body weight distribution measurements were taken for each subject while (1) the mandible was in the rest position (no tooth contact) (RES), (2) subject was clenching (maximum intercuspation of the teeth with heavy occlusal forces) (CL), (3) subject was clenching on the right side (with 1 mm disocclusion on the left side) (CLR), and (4) subject was clenching on the left side (with 1 mm disocclusion on the right side) (CLL). The lateral and the anteroposterior body weight distribution changes during the different clenching conditions (both sides, right, and left) were compared to those at which the mandible was at the rest position. The statistical significance of these results was tested with a Chi-Squared test (p<0.05). Based on the findings of the present study it was concluded that clenching and occlusal instability are associated with lateral body weight distribution changes.

## 1. Introduction

The stomatognathic system is a complex and highly refined unit consisting of the teeth, the temporomandibular joint, and the neuromuscular mechanism. The role of dental occlusion in temporomandibular disorders has been extensively discussed in the dental literature [1–6]. Moreover, it has been suggested that occlusal disharmony or clenching influences the activity of the sternocleidomastoid and trapezius muscles and therefore affects neck position [7, 8]. Recently acquired data indicate that dental occlusion can also contribute to postural changes [9–11]. On the other hand, changes of the body's muscular stability can also influence mandibular position [12–16].

It has been hypothesized that clenching has a direct effect on the posture of both the trunk and the lower limb [17]. There is some documentation for subjects with primary and early permanent dentition [18], as well as for patients presenting with temporomandibular disorders [16, 19]. Additionally, there is some recently published research supporting the notion that there is no detectable [20–22] or weak [9, 10] correlation between dental occlusion and body posture. The contradicting results, as well as the fact that the instrumentation employed in past research projects [17, 23] could not provide very accurate information regarding the body weight distribution on the soles, necessitate further investigation into this area. The purpose of this study was to investigate whether or not clenching and occlusal instability in Angle's Class I malocclusion healthy adult subjects have an effect on body weight distribution. For this research, a system that has integrated paper thin sensors and detects, displays, and records plantar forces was employed. The null hypothesis was that clenching and occlusal instability artificially created in Class I malocclusion healthy adult subjects have no effect on lateral body weight distribution at the feet. Additionally, clenching and occlusal instability artificially created in Class I malocclusion healthy adult subjects have no effect on anteroposterior body weight distribution at the feet level.

## 2. Materials and Methods

**Figure 1:**
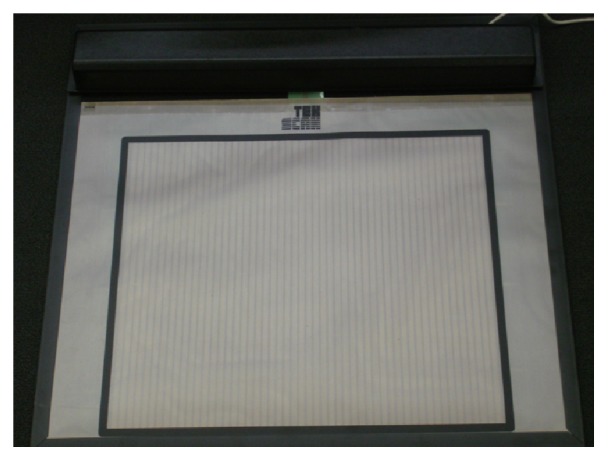
The postural platform (MatScan, Tekscan Inc., Boston, MA, USA) used for the purposes of this study.

**Figure 2:**
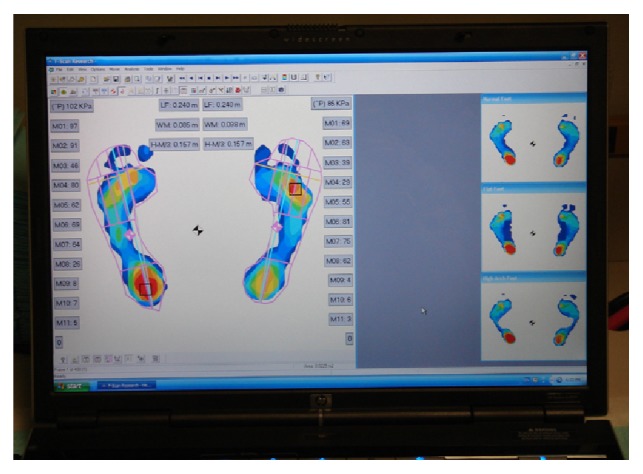
The personal computer used for data storage and analysis.

Twenty adults (fourteen males and six females, ages 27-40, mean age 31.7 years, SD 3.32) who were residents and/or faculty of the Tufts University School of Dental Medicine participated in this study [24]. The sample size was set according to previous research in that field [25–27]. Additionally, Perinetti and Contardo [21] have determined that 17 subjects is the sample size needed to detect an ES coefficient of 1.0 with a power of 0.80 and an *α* equal to 0.05. All subjects were required to read and sign a consent form approved by the Institutional Review Board (#8121). The subjects had no missing teeth, no signs of temporomandibular disorders, and no history of neurologic or musculoskeletal disease. Other criteria for the participation in the study included absence of onlays and absence of anterior and/or posterior crossbite. A full permanent dentition with an Angle's Class I occlusal relationship was required. Subjects' dental occlusion was evaluated in order for stable occlusal contacts and absence of any pathology that could have altered the outcome of this research to be confirmed. All subjects avoided heavy exercise and consumption of alcoholic beverages for at least 24 hours prior to their participation in the study. The pressure assessment system (MatScan, Tekscan Inc., Boston, MA, USA) (Figure 1) consisted of a large postural platform sensor (432 mm x 368 mm) and a personal computer for data storage and analysis (Figure 2).

**Figure 3:**
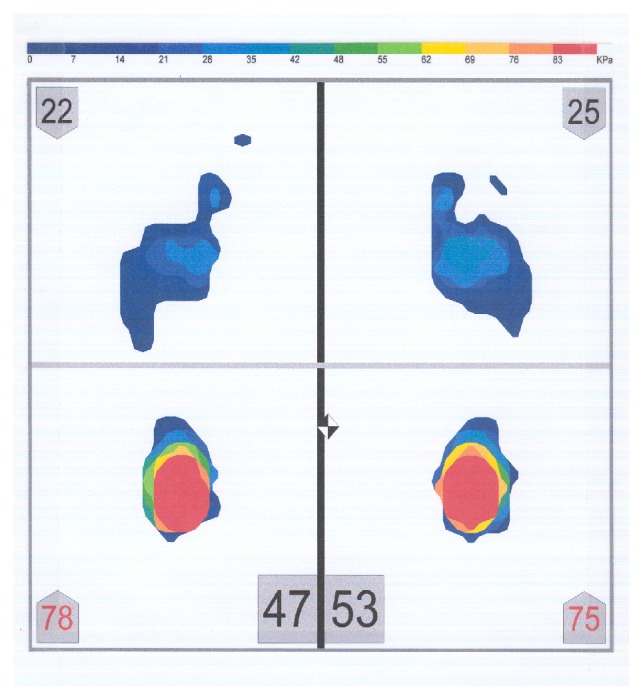
Example of a body weight distribution while the mandible of a subject was in the rest position (no tooth contact) (RES).

**Figure 4:**
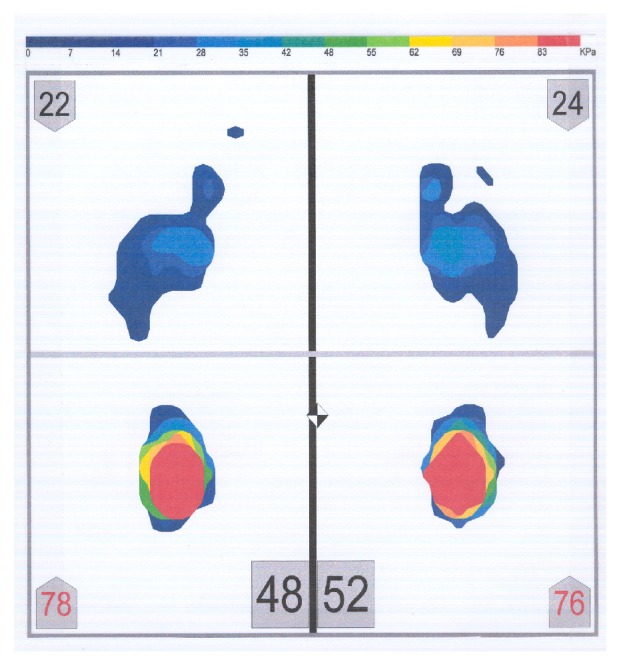
Example of a body weight distribution while a subject was clenching (maximum intercuspation of the teeth, with heavy occlusal forces) (CL).

**Figure 5:**
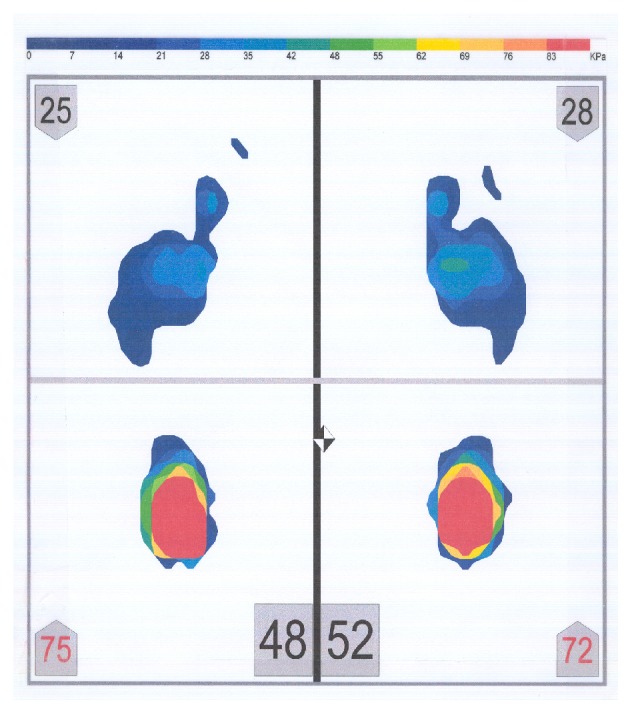
Example of a body weight distribution while a subject was clenching on the right side (with 1 mm disocclusion on the left side) (CLR).

**Figure 6:**
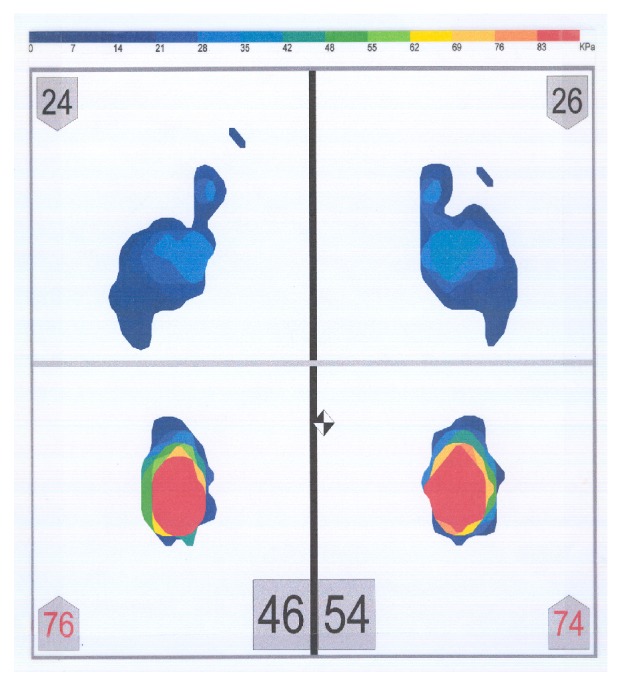
Example of a body weight distribution while a subject was clenching on the left side (with 1 mm disocclusion on the right side) (CLL).

**Figure 7:**
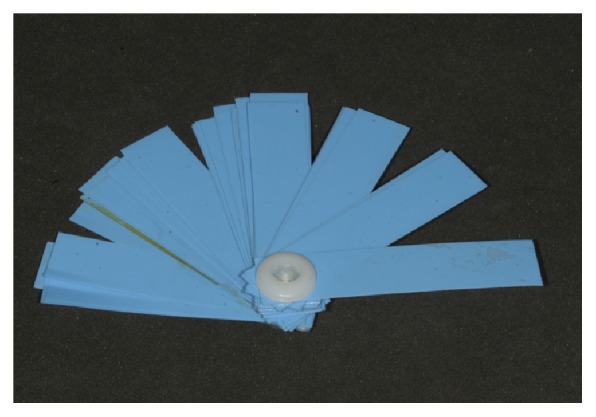
A disposable leaf gage used to create a space between the maxillary and the mandibular dental arches.

The postural platform consisted of several layers of electrically conductive ink rows and columns on a polyester film sheet. The rows and columns formed an X-Y grid of 2288 sensing elements, giving a spatial resolution of 1.4 sensors/cm^2^. The system was calibrated before each subject's measurement, according to the manufacturer's instructions. The measuring instrument was placed, but not fixed, on a hard, flat surface. Each subject stood barefoot on the sensor mat in a comfortable stance with their arms relaxed beside their trunk. Subjects were asked to stand as still as possible and maintain this relaxed head and body posture. During the measurement period, subjects were asked to look at a mark on a wall, at eye level. The mark had a diameter of 1 cm. The distance between the subjects and the wall was 2 meters. Subjects were not allowed to move their feet during the test. Four body weight distribution measurements were taken for each subject while (1) the mandible was in the rest position (no tooth contact) (RES) (Figure 3), (2) subject was clenching (CL) (Figure 4), (3) subject was clenching on the right side (with 1 mm disocclusion on the left side) (CLR) (Figure 5), and (4) subject was clenching on the left side (with 1 mm disocclusion on the right side) (CLL) (Figure 6). The 1mm disocclusion on each side was achieved by means of a disposable leaf gage which was placed on the contralateral (clenching) side (Figure 7).

Each measurement lasted 8 seconds, while a total of 400 frames were collected in each measurement. Thus, the body weight distribution was recorded every 0.02 seconds. The overall lateral body weight distribution (OLWD) and the anteroposterior body weight distribution (APWD) were then calculated using the following equations:

(1) OLWD%=50−left  body  weight  percentage  valueAPWD%=50−posterior  left  body  weight  percentage  value.

The last equation provided the body weight distribution for each foot separately.

When the body weight distribution was equal bilaterally, OLWD was zero. When the body weight was shifted to the right, OLWD was positive. When the body weight was shifted to the left, OLWD was negative. Similarly, when the body weight distribution was equal anteroposteriorly, APWD was zero. When the body weight was shifted anteriorly, APWD was positive. When the body weight was shifted posteriorly, APWD was negative.

To investigate whether the body weight distribution changed during the different clenching conditions, the OLWD and the APWD during clenching (both sides, right, and left) were compared to those at which the mandible was at the rest position. The number of subjects who changed their body weight distribution in the different occlusal conditions was calculated. The side where the body weight distribution shifted to was classified. The anteroposterior body weight shift was classified as well. The subjects were categorized relative to the lateral and anteroposterior side where the body weight distribution shifted when clenching in each occlusal condition and is then calculated. To investigate whether the different occlusal conditions (clenching on both sides, clenching on the right side, and clenching on the left side) had a statistically significant impact on the body weight distribution, a Chi-Squared test (p<0.05) was performed.

The research data were summarized by presenting counts and proportions (%) relative to the distributions of the studied subjects' classifications. The association between occlusal condition and body weight distribution was examined by a modified version of the Chi-Squared test, proposed by Decady and Thomas [28]. This test is appropriate in cases where the available cross-tabulations are filled with multiple-response data. In order to depict (visualize) the inherent constructs of the statistically significant associations (correlations), the Correspondence Analysis (CA) method [29] was applied. The significance level of the statistical tests was predetermined at *a* = 0.05. The basic statistical analyses were performed using the statistical package SPSS ver. 11.5. The CHIC Analysis software was used for the application of the CA method. A special spreadsheet was developed in Excel to enable the application of the modified version of the Chi-Squared test. The classical Pearson Chi-Squared test of association fails for two reasons. First, the use of the marginal totals from multiple-response tables of the type shown in Tables 1 and 2 leads to inappropriate expected values and corresponding estimates [30, 31]. Second, multiple responses from the same individual violate the standard assumption of independence of observations. Thus, in order to study the significance of the association between occlusal condition and body weight distribution, a modified and corrected version of the Chi-Squared test, proposed by Decady and Thomas [28], was applied.

## 3. Results

**Figure 8:**
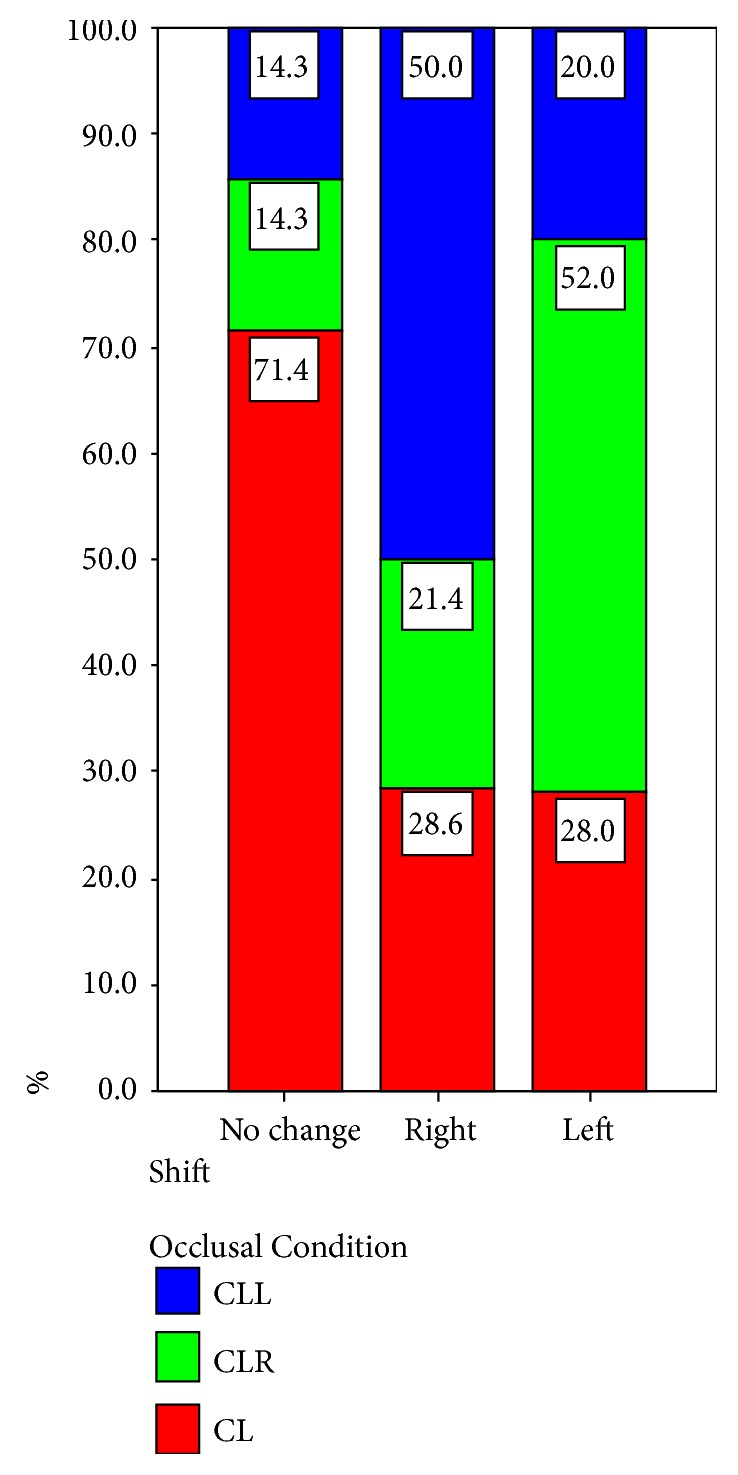
Comparison of shift in relation to the distribution of the occlusal condition (lateral body weight distribution).

**Figure 9:**
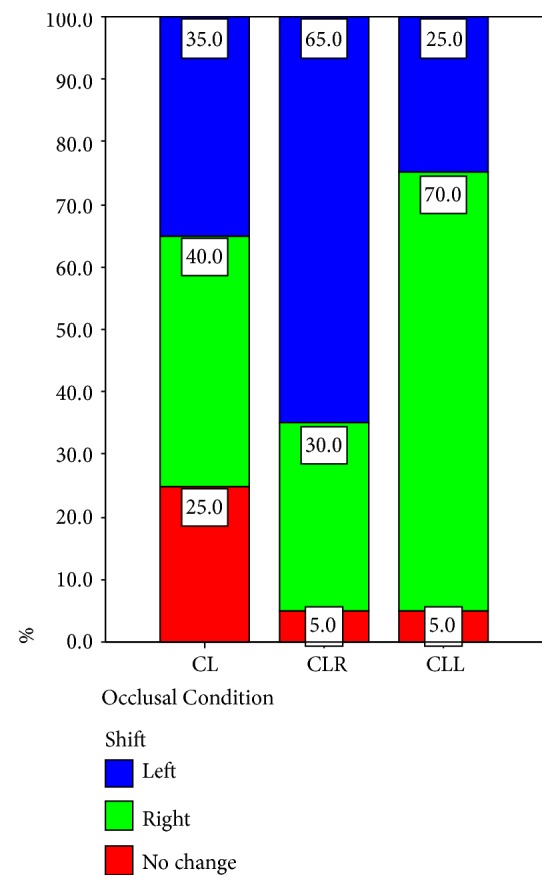
Comparison of occlusal condition in relation to the distribution of the shift (lateral body weight distribution).

**Figure 10:**
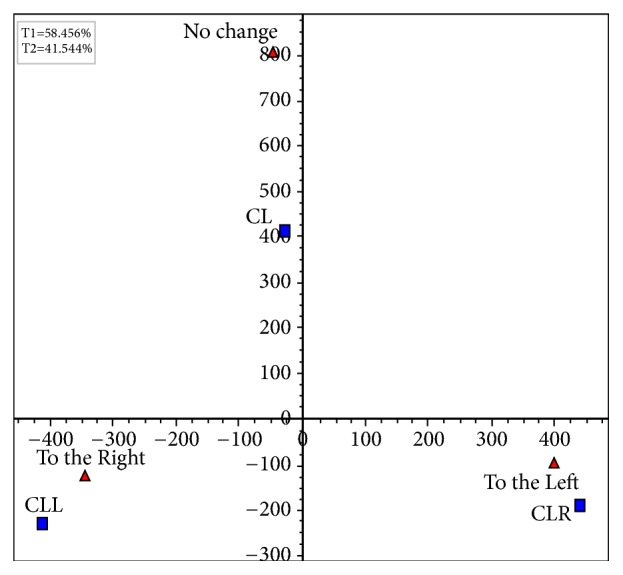
The factorial plane 1 x 2.

The results of the comparison between the lateral body weight distribution before and during clenching are shown in Table 1. Five subjects (25%) (3 male and 2 female) did not show any shift during clenching on both sides, while eight subjects (40%) showed a shift to the right foot and seven subjects (35%) demonstrated a shift to the left. When data on clenching on the right side were analyzed, the following observations were made: one subject (5%) (male) did not show any shift of the body weight distribution; six subjects (30%) demonstrated a shift to the right while thirteen subjects (65%) showed a shift to the left. When data on clenching on the left side were analyzed, we observed that one subject (5%) (male) did not show any shift of the body weight distribution; fourteen subjects (70%) demonstrated a shift to the right, while five subjects (25%) showed a shift to the left. One of the subjects, who did not have any shifting during clenching on both sides, did not show any body weight distribution shifting during clenching on the left side either (Tables 1 and 3, Figures 8–10).

**Figure 11:**
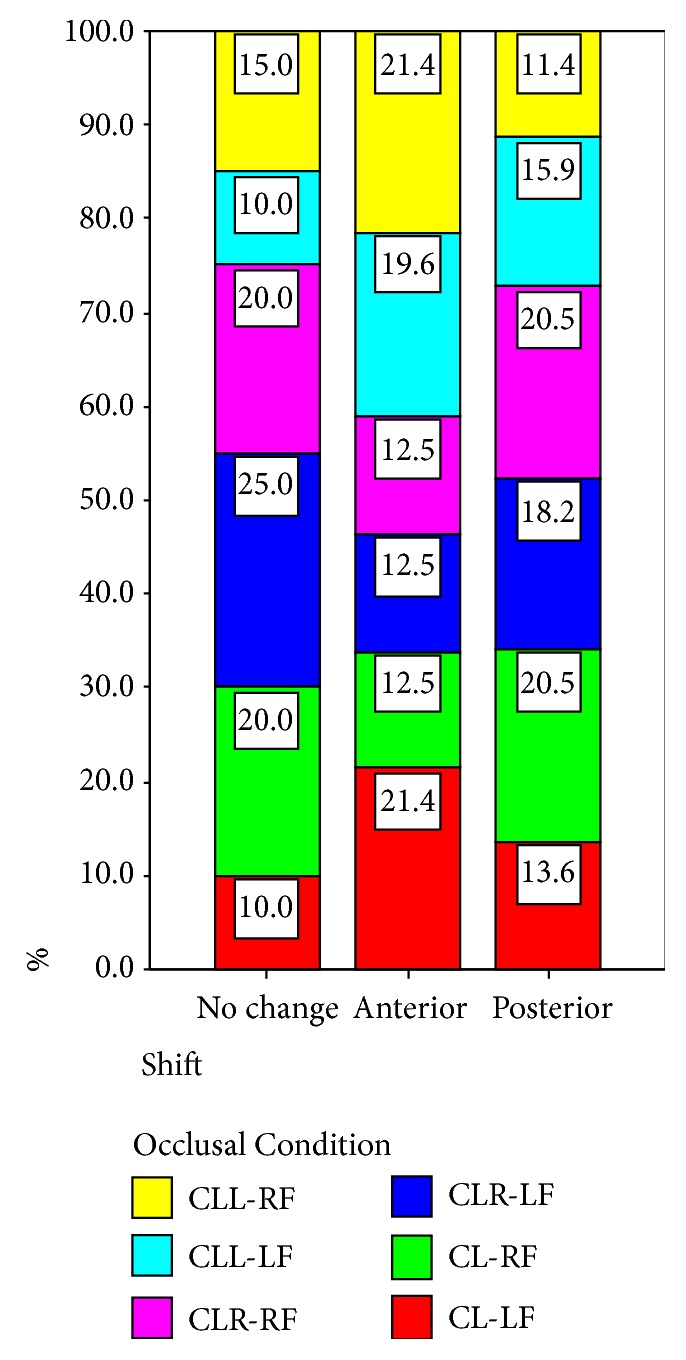
Comparison of shift in relation to the distribution of the occlusal condition (anteroposterior body weight distribution).

**Figure 12:**
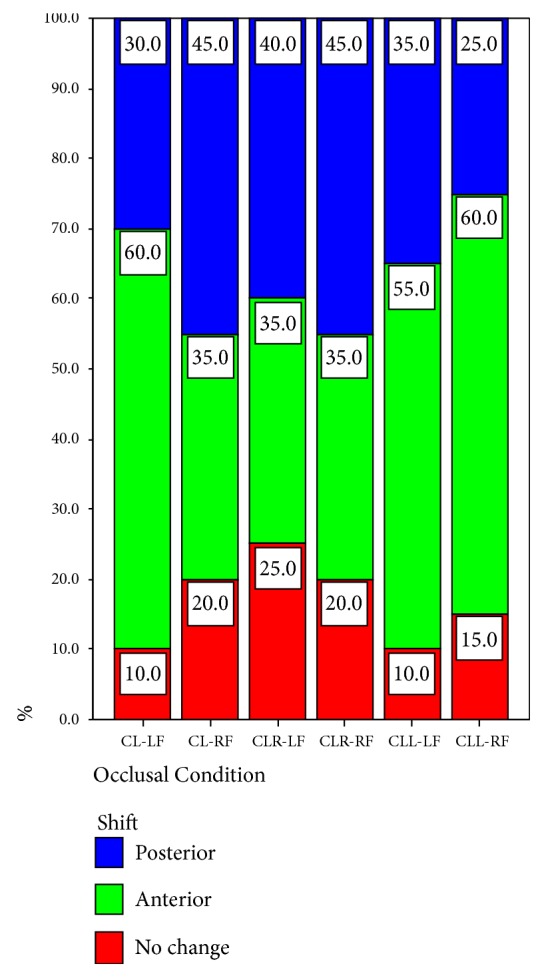
Comparison of occlusal condition in relation to the distribution of the shift (anteroposterior body weight distribution).

The results of the comparison between the anteroposterior body weight distribution before and during clenching are shown in Table 2. These results are presented for both the left (LF) and the right (RF) foot. The data collected for the left foot indicated that two of the subjects (10%) did not show any alteration in the anteroposterior body weight distribution; twelve (60%) showed an anterior body weight shift, while six (30%) demonstrated a posterior body weight shift during clenching on both sides of the dental arch. The data collected for the right foot and for the same occlusal condition also indicated that four subjects (20%) did not show any alteration in the anteroposterior body weight distribution; seven (35%) showed an anterior body weight shift, while nine (40%) demonstrated a posterior body weight shift during clenching on both sides (Tables 2 and 4, Figures 11 and 12).

The research data of the left foot indicated that five of the subjects (25%) did not show any alteration in the anteroposterior body weight distribution; seven (35%) showed an anterior body weight shift, while eight (40%) demonstrated a posterior body weight shift during clenching on the right side of the dental arch. The data of the right foot and for the same occlusal condition also indicated that four subjects (20%) did not show any change in the anteroposterior body weight distribution; seven (35%) showed an anterior body weight shift, while nine (45%) demonstrated a posterior body weight shift during clenching on the right side.

Two of the subjects (10%) did not show any anteroposterior body weight shift to the left foot; eleven (55%) showed an anterior body weight shift, while seven (35%) demonstrated a posterior body weight shift during clenching on the left side of the dental arch. Three subjects (15%) did not show any anteroposterior body weight distribution changes to the right foot (for the same occlusal condition), twelve (60%) showed an anterior body weight shift, and five (25%) demonstrated a posterior body weight shift during clenching on the left side.

It is evident that the data presented in Tables 1 and 2 are multiple-response data. The modified version of the Chi-Squared test in Table 1 revealed a statistically significant association between occlusal condition and lateral body weight distribution (modified *χ*_*m*_^2^(6) = 12.446, correction factor *δ* = 0.667, corrected *χ*_*c*_^2^(6) = 18.669, *p* = 0.005). In order to depict the association between occlusal condition and body weight distribution, the Correspondence Analysis method [29] was applied (Table 1). The factorial plane 1×2 is presented in Figure 10. It can be observed that CLL is associated with “to the right” and contradicts, on the first-horizontal axis, to CLR which is linked to “to the left”. The CL is related to “no change” independently from the others, since these two points (CL and “no change”) load mainly to the second factorial axis (vertical axis). Based on the data presented in Table 2, the modified version of the Chi-Squared test did not reveal a statistically significant association between occlusal condition and anteroposterior body weight distribution (modified *χ*_*m*_^2^(15) = 7.590, correction factor *δ* = 0.667, corrected *χ*_*c*_^2^(15) = 11.384, *p* = 0.725).

## 4. Discussion 

The results of the present study support the notion that clenching and occlusal instability are associated with lateral body weight distribution changes. Therefore, the first part of the null hypothesis should be rejected.

The lateral body weight distribution of the majority of the participating subjects changed during clenching, with the masseter and temporal muscles being active during that period. A very important finding of the present study is that the subjects shifted their body weight opposite to the clenching side. It should be mentioned that the neck muscles (especially the sternocleidomastoid and the trapezius) demonstrate an increased activity during clenching [7, 8, 32]. Absence of posterior occlusal support results in an alteration of the information sent from the periodontal ligament mechanoreceptors as well as of the information sent from masticatory muscles or temporomandibular joint proprioceptors. These changes can affect the cervical muscles through the trigeminal nerve [33]. It has also been suggested that the activity of the sternocleidomastoid muscle—while subjects were performing stomatognathic functions—was due to the reflex activity resulting from the stretch reflex and central control related to the trigeminal nerve [34]. This results in changes of the head position due to stomatognathic function. Additional research [35, 36] on this field verified these findings and concluded that cervical muscles balance and affect the head position in order for smoother jaw movements to be achieved. It should be mentioned that control of the head posture is primarily achieved by cervical nerves C1 and C4 [37]. The neck muscles affect the head posture by submitting proprioceptive inputs. A stable head position is essential for the control of body posture. This mechanism is very important for the maintenance of the postural balance [38]. Likewise, the spinal column and the lower limbs are responsible for the body posture. The sense of equilibrium and the antigravity (postural) muscles also assist the maintenance of the body posture [39].

A lateral shift of the body weight distribution during clenching, observed in the present study, was probably caused in order to maintain an upright posture to prevent falling with the antigravity muscles controlling, and the spinal column and the lower limbs bending, since the head center of mass shifted due to the activation of the neck muscles. This interesting theory has been suggested by Yoshino et al. [23]. The subjects tended to change the head position laterally towards the clenching side of the dental arch. As a result, the body weight distribution of the trunk and the lower limbs shifted towards the opposing side of the clenching, in order to compensate for the initial altered head position. This fact has also been demonstrated by previous researchers [9, 10, 40, 41]. It has been reported [23] that if, due to occlusal instability, the lateral shift in the body weight distribution is repeated for hours, the antigravity muscles, which are related to maintaining standing posture, will become hypertonic. As a result, the subject will get muscle pain and additionally all antigravity muscles will become inharmonious. It can therefore be concluded that the neck muscles can become inharmonious due to unilateral or bilateral loss of occlusal support. As a result, the body posture may be affected and altered to an abnormal position [23]. This can then cause neck and shoulder pain. It should be mentioned however that body posture is attained by the sense of equilibrium which consists of vestibule, somatic, and visual sensation [39]. Stimulation of the vestibular system by changing head position has a descending influence on the triceps muscle of the calf and on the soleus muscle, which are both antigravity muscles [42].

The present study did not investigate any head postural changes due to occlusal instability and clenching. Occlusal instability (clenching on one side) has probably caused a disharmony between bilateral masticatory muscles. This disharmony may have also affected the bilateral activity of the sternocleidomastoid muscles [23, 43–46]. The disharmony of the masticatory and the sternocleidomastoid muscles may have caused an alteration of the proprioceptive inputs in these muscles. Additionally, the fact that occlusal contacts occurred in only one side of the dental arch may have caused a change in the afferent information coming from the periodontium. As a result, the bilateral disharmony of the neuromuscular system may have caused an instability of the head position and consequently of the body posture which was detected during this study [46].

The present study could not establish a correlation between clenching and occlusal instability and anteroposterior body weight distribution changes. Therefore, the second part of the null hypothesis should be accepted.

Although the first part of this study (lateral body weight changes) is in accordance with the findings of Yoshino et al. [23] and Sakaguchi et al. [46], the second part (anteroposterior body weight changes) is in contrast with those studies. The second part of the study is in accordance with the findings of Perinetti [20] and Perinetti et al. [21], who did not find a detectable correlation between dental occlusion and posture. Although a tendency for an anterior body weight displacement could be observed, a statistically significant difference could not be established. It should be mentioned however that, in both Yoshino's [23] and Sakaguchi's [46] studies, the Angle's classification of the subjects is not reported. On the other hand, all the subjects who participated in Perinetti's study [20] were classified as having an Angle's Class I relationship. Likewise, the subjects in the present study had an Angle's Class I molar relationship. It has been reported [47–49] that there is a correlation between occlusal classes of malocclusion and head postural attitudes. In that manner there is a possibility that the subjects who participated in the present study did not correlate to an anterior postural attitude. Additional parameters that may affect the study results may include repeatability of measurements, sample size, age, gender, and time of clenching or exposure to occlusal instability. Use of different equipment recording body posture under static and dynamic condition between the present and previous studies may have also influenced the final outcomes. Further clinical research is needed in this area in order to establish definitive conclusions.

## 5. Conclusions

Within the limitations of the present study, the following conclusions can be drawn:

1. Clenching and occlusal instability are associated with lateral body weight distribution changes.
2. Clenching and occlusal instability are not associated with anteroposterior body weight distribution changes.

## Figures and Tables

**Table 1:** Distribution of subjects (N=20) classified on the lateral side where the body weight distribution was shifted by clenching (*p*=0.005).

SHIFT	CL	CLR	CLL	TOTAL
No Change	5	1	1	7
To the RIGHT	8	6	14	28
To the Left	7	13	5	25
Total	20	20	20	60

**Table 2:** Distribution of subjects (N=20) classified on the anteroposterior side where the body weight distribution was shifted by clenching (p=0.725).

SHIFT	CL-LF	CL-RF	CLR-LF	CLR-RF	CLL-LF	CLL-RF	TOTAL
No Change	2	4	5	4	2	3	20
To Anterior	12	7	7	7	11	12	56
To Posterior	6	9	8	9	7	5	44
Total	20	20	20	20	20	20	120

**Table 3:** Shift *∗* occlusal condition crosstabulation (lateral body weight distribution) % within occlusal condition (p=0.005).

	Occlusal Condition			Total
	1 CL	2 CLR	3 CLL	
Shift				
1 No change	25.0%	5.0%	5.0%	11.7%
2 Right	40.0%	30.0%	70.0%	46.7%
3 Left	35.0%	65.0%	25.0%	41.7%
Total	100.0%	100.0%	100.0%	100.0%

**Table 4:** Shift *∗* occlusal condition crosstabulation (anteroposterior body weight distribution) % within occlusal condition (p=0.725).

	Occlusal Condition						Total
	1 CL-LF	2 CL-RF	3 CLR-LF	4 CLR-RF	5 CLL-LF	6 CLL-RF	
Shift							
1 No change	10.0%	20.0%	25.0%	20.0%	10.0%	15.0%	16.7%
2 Anterior	60.0%	35.0%	35.0%	35.0%	55.0%	60.0%	46.7%
3 Posterior	30.0%	45.0%	40.0%	45.0%	35.0%	25.0%	36.7%
Total	100.0%	100.0%	100.0%	100.0%	100.0%	100.0%	100.0%

## Data Availability

All data supporting the results reported are available in technical report that has been composed and is available from the corresponding author upon request.
